# Progress Toward Poliomyelitis Eradication ― Afghanistan, January 2021–September 2022

**DOI:** 10.15585/mmwr.mm7149a1

**Published:** 2022-12-09

**Authors:** Abdinoor Mohamed, Irfan Elahi Akbar, Sumangala Chaudhury, Mufti Zubair Wadood, Fazal Ather, Jaume Jorba, Maureen Martinez

**Affiliations:** ^1^Global Immunization Division, Center for Global Health, CDC; ^2^Polio Eradication Department, World Health Organization, Kabul, Afghanistan; ^3^Polio Eradication Department, World Health Organization, Geneva, Switzerland; ^4^Polio Eradication Department, World Health Organization, Amman, Jordan; ^5^Division of Viral Diseases, National Center for Immunization and Respiratory Diseases, CDC.

Afghanistan and Pakistan are the two remaining countries with endemic wild poliovirus type 1 (WPV1) transmission (*1*). During 2019–2020, these countries reported their highest numbers of WPV1 cases since 2014 and experienced outbreaks of type 2 circulating vaccine-derived poliovirus (cVDPV2) (*2*–*4*).* In Afghanistan, the number of WPV1 cases nearly doubled, from 29 in 2019 to 56 in 2020; 308 cVDPV2 cases were reported during 2020. After years of active conflict, the Afghanistan government was fully replaced by the Taliban de facto government on August 15, 2021. This report describes activities and progress toward polio eradication in Afghanistan during January 2021–September 2022 and updates previous reports (*3*,*4*). During January–December 2021, four WPV1 and 43 cVDPV2 cases were detected, representing decreases of 93% from 56 cases and 86% from 308 cases, respectively, during 2020. During January–September 2022 (reported as of October 20), two WPV1 cases and zero cVDPV2 cases were detected. Although no supplementary immunization activities (SIAs)^†^ occurred during July–October 2021, SIAs resumed during November 2021 in all districts after the political transition, and 3.5–4.5 million previously unreachable persons have been vaccinated since. However, restrictions on how SIAs are conducted are still in place in the critical South Region provinces of Kandahar, Helmand, and Uruzgan. If efforts to vaccinate all children are enhanced and expanded, Afghanistan has an opportunity to interrupt WPV1 transmission during 2023.

## Immunization Activities

The World Health Organization (WHO) and UNICEF estimate of national 2021 immunization coverage with 3 doses of oral poliovirus vaccine (OPV3) among children aged 12–23 months was 71% compared with 75% in 2020. The estimated 1-dose coverage with injectable inactivated poliovirus vaccine was 67% in 2021 compared with 62% in 2020 (*5*). However, these national estimates obscure substantial subnational coverage gaps.

Because of the low quality of routine immunization (RI) data, caregiver recall dose history from investigations of acute flaccid paralysis (AFP) in children who do not have laboratory evidence of poliovirus infection (nonpolio AFP [NPAFP]) is used as a proxy for RI coverage. Among the 2,567 infants and children aged 6–59 months with NPAFP in 2021, 68% had received ≥3 RI OPV doses nationwide; 17% had not received any RI dose. In 2022, as of August 31, these percentages were similar (67% and 17%, respectively). In 2021, 4% of children with NPAFP had not received any OPV through RI or SIAs (zero-dose children); the percentage of zero-dose children declined to 2% in 2022. By province, the highest percentages of zero-dose children in 2021 were reported from Zabul (28%) and Helmand (17%) provinces in the South Region, and from Nuristan (11%) in the East Region in 2022, as of August 31.

In 2015, WHO declared wild poliovirus type 2 to be eradicated.^§^ In 2016, Afghanistan joined OPV-using countries around the world in implementing a synchronized withdrawal of trivalent OPV (tOPV), containing Sabin-strain types 1, 2, and 3, and replacement with bivalent OPV (bOPV), containing Sabin-strain types 1 and 3, and ≥1 dose of inactivated poliovirus vaccine as part of containment efforts for all type 2 polioviruses (*6*). However, in 2020 when Afghanistan began to report both cVDPV2 and WPV1 polio cases, the Global Polio Eradication Initiative authorized the use of tOPV for outbreak response. During January–December 2021, five SIAs were conducted, including two after the political transition; all were national immunization days (NIDs). During January–September 2022, nine SIAs targeting children aged <5 years were conducted: six NIDs, one subnational immunization day, and two large-scale SIAs conducted in response to a polio case (case-response SIAs). The NIDs conducted in January, March, November, and December 2021 used tOPV; the remaining SIAs in 2021 and 2022 used bOPV.

In November 2021, during the first SIA after the political transition, only 53% of the 10 million children targeted by SIAs lived in areas without any restriction on how SIAs were conducted. This percentage gradually increased and reached 76% by the September 2022 NID. The reported NID OPV coverage in areas where SIAs were conducted without restrictions increased from 72% in June 2021 to approximately 100% in March, May, and June 2022, although this figure likely overestimates true coverage because of poor target setting and data management. Reported coverage was <50% in districts with restrictions on SIA implementation. In 2022, to date, the program has reached 3.5–4.5 million children previously unreachable because the insurgency prevented access before the government transition.

Lot quality assurance sampling (LQAS)^¶^ surveys are conducted to assess SIA quality. Previously limited in implementation, these surveys were expanded nationwide in 2022 to include all districts. During the first five 2022 NIDs, 1,319 lots were assessed, a 65% increase over the 797 assessed during 2021, and the quality of implementing LQAS surveys improved. More lots were reported as failed at the 90% SIA coverage threshold in 2022 (37%) than in 2021 (18%); however, the primary reason was that approximately all lots in districts where SIA implementation methods were restricted failed LQAS assessments.

## Poliovirus Surveillance

**AFP surveillance.** The Afghanistan AFP surveillance network comprises 1,947 active surveillance sites (visited by surveillance officers), 3,222 zero-reporting sites with passive monthly reporting, and 46,000 community-based reporting volunteers. Detection of two or more NPAFP cases per 100,000 persons aged <15 years** along with collection of adequate stool specimens^††^ from ≥80% of AFP cases indicates that surveillance is sufficiently sensitive to detect a case of paralytic polio. During 2021, the national NPAFP rate was 19 per 100,000 persons aged <15 years (regional range = 12–26); during January–August 2022, the annualized NPAFP rate was 25 (regional range = 17–40). The percentage of AFP cases with adequate specimens was 94% in 2021 and 95% to date in 2022 (regional range = 90%–98% in 2021 and 92%–98% in 2022) (Table).

**TABLE:** Acute flaccid paralysis surveillance performance indicators, reported cases of wild poliovirus and vaccine-derived poliovirus type 2,* and number of environmental samples with detection of wild poliovirus type 1, by region and period — Afghanistan, January 2021–September 2022^†^

Region	AFP surveillance performance indicators						No. of cases reported						No. of ES samples with WPV1 detected^§^		
							WPV1			cVDPV2					
	No. of AFP cases		NPAFP rate^¶^		% of cases with adequate stool specimens**		2021		2022	2021		2022	2021		2022
	2021	2022	2021	2022^††^	2021	2022	Jan–Jun	Jul–Dec	Jan–Sep	Jan–Jun	Jul–Dec	Jan–Sep	Jan–Jun	Jul–Dec	Jan–Sep
**All**	**4,088**	**3,580**	**18.7**	**24.8**	**94.0**	**94.9**	**1**	**3**	**2**	**42**	**1**	**0**	**1**	**0**	**3**
**Badakhshan**	90	102	14.2	24.1	95.6	96.1	0	0	0	0	0	0	0	0	0
**Central**	843	612	17.1	18.6	98.2	97.7	0	0	0	3	1	0	0	0	0
**East**	573	577	26.4	39.8	95.5	94.5	0	0	1	0	0	0	0	0	3
**North**	329	313	12.0	17.4	90.6	93.5	0	0	0	2	0	0	0	0	0
**Northeast**	408	348	16.8	21.5	93.9	93.9	0	3	0	0	0	0	0	0	0
**South**	827	721	21.8	28.7	89.7	91.9	0	0	0	12	0	0	1	0	0
**Southeast**	388	364	17.7	25.2	96.1	97.5	1	0	1	8	0	0	0	0	0
**West**	630	543	21.1	28.0	93.2	95.4	0	0	0	17	0	0	0	0	0

**Abbreviations:** AFP = acute flaccid paralysis; cVDPV2 = type 2 circulating vaccine-derived poliovirus; ES = environmental surveillance; NPAFP = nonpolio acute flaccid paralysis; WPV1 = wild poliovirus type 1.

* cVDPVs are genetically linked VDPV2 isolates for which there is evidence of person-to-person transmission in the community.

^†^ Data as of October 20, 2022.

^§^ Total number of ES samples by period, January 2021–September 2022.

^¶^ Cases per 100,000 persons aged <15 years. The surveillance performance indicator target is two or more NPAFP cases per 100,000 persons aged <15 years per year.

** Adequate stool specimens are defined as two stool specimens of sufficient quality for laboratory analysis, collected ≥24 hours apart, both within 14 days of paralysis onset, and arriving in good condition at a World Health Organization–accredited laboratory with reverse cold chain maintained, without leakage or desiccation, and with proper documentation.

^††^ Annualized from AFP surveillance data through August 2022.

**Environmental surveillance.** Poliovirus surveillance in Afghanistan is supplemented by environmental surveillance (ES), the systematic sampling and virologic testing of sewage at 30 sites in 13 provinces. In 2021, only one WPV1 sample was detected among ES samples from the Helmand province in the South Region. As of October 20, 2022, WPV1 was detected during the reporting period in three sites: one in Nangarhar and two in Kunar provinces in the East Region, with the latest from a site in Kunar province in September 2022. During 2021, cVDPV2 was isolated from 40 ES specimens from Helmand, Herat, Kabul, Kandahar, Kunduz, and Nangarhar provinces; the latest isolation was from an ES sample collected in June 2021 in Kandahar province.

## Epidemiology of Polio Cases and Genomic Sequence Analysis of Poliovirus Isolates

**FIGURE 1:**
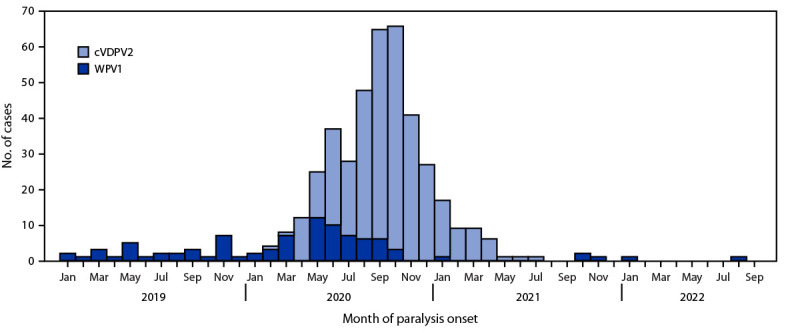
Number of wild poliovirus type 1 cases and circulating vaccine-derived poliovirus type 2*^,^^†^ cases, by month of onset of paralysis — Afghanistan, January 2019–September 2022^§^ **Abbreviations:** cVDPV2 = circulating vaccine-derived poliovirus type 2; WPV1 = wild poliovirus type 1. * The number of cases of WPV1 and cVDPV2 were 90 and 351, respectively. ^†^ cVDPVs are genetically linked VDPV2 isolates for which there is evidence of person-to-person transmission in the community. ^§^ Data as of October 20, 2022.

**FIGURE 2:**
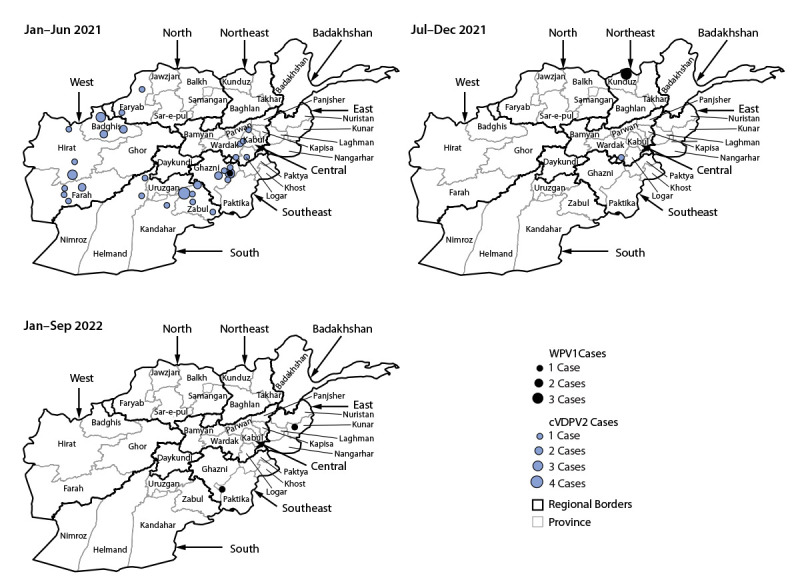
Cases of polio caused by wild poliovirus type1 and circulating vaccine-derived poliovirus type 2,* by province and period — Afghanistan, January 2021–September 2022^†^^,^^§^ **Abbreviations:** cVDPV2 = circulating vaccine-derived poliovirus type 2; WPV1 = wild poliovirus type 1. * cVDPVs are genetically linked VDPV2 isolates for which there is evidence of person-to-person transmission in the community. ^†^ Total cases by period: January–June 2021 = one WPV1 and 42 cVDPV2, July–December 2021 = three WPV1 and one cVDPV2, and January–September 2022 = two WPV1 and zero cVDPV2. ^§^ Data as of October 20, 2022.

Four WPV1 cases were detected in 2021, one from Ghazni province in the Southeast Region and three from Kunduz province in the Northeast Region. During January–September 2022 (as of October 20, 2022), only two WPV1 cases were reported in two regions: one each from Paktika (Southeast Region) and Kunar provinces (East Region) (Table) (Figure 1) (Figure 2). All six patients in 2021 and 2022 were aged 10–25 months; three had never received OPV through RI services but had received 1 SIA dose, one had never received any OPV, one reportedly received 2 RI doses and 1 SIA dose, and one reportedly received 4 RI doses and 7 SIA doses.

Genomic sequence analysis of the region encoding the viral capsid protein 1 (VP1) of poliovirus isolates provided evidence of cross-border transmission between Afghanistan and Pakistan during 2019–2022, with sustained local transmission in both countries. During January 2021–September 2022, four of six WPV1 isolates from AFP patients and one of four WPV1 ES isolates from Afghanistan had their closest genetic links to WPV1 isolates from Pakistan. The poliovirus isolated from the first case during 2022, reported from Paktika province (Southeast Region), was genetically linked to previous circulation in Pakistan’s Baluchistan province. The poliovirus from the second 2022 case, reported from Kunar province (East Region), was genetically linked with transmission in the Northeast Region of Afghanistan. During January 2021–September 2022, only two WPV1 genetic clusters (groups of viruses sharing ≥95% VP1 sequence identity) were detected among AFP cases and environmental samples. Of the five WPV1 viruses detected in 2021, two (40%) were orphan viruses,^§§^ indicating possible gaps in surveillance; no orphan viruses have been detected in 2022 to date.

Among 43 cVDPV2 cases reported in 2021, 29 (67%) were in the PAK-GB-1 emergence group, first detected in Gilgit-Baltistan, Pakistan, and 14 were in the AFG-NGR-1 emergence group, first detected in Afghanistan’s Nangarhar province (*3*). Paralysis onset in the patient with the most recently detected cVDPV2 case was in July 2021.

## Discussion

After confirmation of large numbers of both WPV1 and cVDPV2 polio cases during 2019–2020 in Afghanistan and Pakistan, both countries jointly reported five WPV1 and 51 cVDPV2 cases in 2021 (*1*). Given that the latest cVDPV2 detection was in Pakistan in August 2021, transmission of cVDPV2 in both countries is likely interrupted. Resurgence of WPV1 cases occurred in 2022 in the south of Khyber Pakhtunkhwa province of Pakistan, an area that directly borders Afghanistan, with substantial social ties and population movement. As of October 20, 2022, Afghanistan has reported two WPV1 cases, one each in the East and Southeast regions. After the political transition, the de facto government’s public health authorities implemented an aggressive SIA schedule during November 2021–September 2022, which resulted in a substantial reduction in the number of unreached children. However, as of September 2022, >85% of children in the South Region where polio is endemic live in areas where restrictions on SIA implementation methods continue.

The findings in this report are subject to at least one limitation. The quality of data on SIA implementation is limited by the low accuracy of reported coverage data. The Global Polio Eradication Initiative is supporting the national program to establish a comprehensive data management system and providing ongoing staff member training.

Current polio eradication efforts in Afghanistan are challenged by a complex humanitarian emergency resulting from the combined impacts of a rapid government transition and a depressed economy, droughts, floods, food insecurity, displacement, and severe gaps in delivery of health services (*7*). In June 2022, a 5.9 magnitude earthquake struck Khost province in the Southeast Region, killing more than 1,000 persons and displacing entire communities (*8*). With progress broadening SIA access since the political transition, the opportunity to end WPV1 transmission in Afghanistan before the end of 2023 appears to be attainable. Ending transmission, however, depends on continued and expanded SIAs throughout the country, including in the high-risk provinces of the South Region.

**Summary**

**What is already known about this topic?**

Afghanistan and Pakistan are the only countries where wild poliovirus type 1 (WPV1) remains endemic.

**What is added by this report?**

Two WPV1 cases had been reported in 2022 as of September 30, compared with one case during the same period in 2021. No type 2 circulating vaccine-derived poliovirus was reported in 2022 compared with 43 cases in 2021. Since the political transition in August 2021, 3.5–4.5 million previously unreachable children were vaccinated; supplementary immunization activity (SIA) restrictions persist in the South Region.

**What are the implications for public health practice?**

Ensuring implementation of high-quality SIAs in all parts of Afghanistan, especially in the high-risk provinces of the South Region, will accelerate progress toward interrupting WPV1 transmission.

## Footnotes

* Vaccine-derived polio viruses can emerge when attenuated oral polio vaccine (OPV) virus reverts to neurovirulence as a result of transmission in areas with low immunization coverage. cVDPV2s are genetically linked VDPV2 isolates for which there is evidence of person-to-person transmission in the community.

† SIAs are mass immunization campaigns intended to supplement the routine immunization systems and target children aged <5 years with OPV, regardless of their vaccination history. In Afghanistan, SIAs are conducted using a variety of methods such as house-to-house, mosque-to-mosque, or site-to-site.

§
https://polioeradication.org/news-post/global-eradication-of-wild-poliovirus-type-2-declared

¶ LQAS is a rapid survey method to assess the quality of vaccination activities after SIAs in predefined areas, such as health districts (lots), using a sample size of 60. LQAS involves dividing the population into lots and ascertaining receipt of vaccination by randomly selecting children within each lot. If the number of unvaccinated children in the sample exceeds three, then the SIA quality in that area is classified as failed (i.e., at a pass threshold of ≥90%) and mop-up activities are recommended. If the threshold of ≥90% is met, the SIA’s quality for the area is classified as having passed.

** NPAFP cases are those that are discarded as not having laboratory or other proof of poliovirus as the cause. The expected background rate of NPAFP is two or more cases per 100,000 children aged <15 years per year, the standard WHO performance indicator target for sufficiently sensitive surveillance to detect one case of polio.

†† Adequate stool specimens are defined as two stool specimens of sufficient quality for laboratory analysis, collected ≥24 hours apart, both within 14 days of paralysis onset, and arriving in good condition at a WHO-accredited laboratory with reverse cold chain maintained, without leakage or desiccation, and with proper documentation. The global standard surveillance performance indicator target is ≥80% of AFP cases with adequate stool specimens collected.

§§ Orphan viruses are ≥1.5% divergent from their closest genetic match (i.e., ≤98.5% identity) and can indicate gaps in AFP surveillance.
